# Acute and Chronic Local Inflammatory Reaction after Implantation of Different Extracellular Porcine Dermis Collagen Matrices in Rats

**DOI:** 10.1155/2015/938059

**Published:** 2015-01-14

**Authors:** Silke Lucke, Andreas Hoene, Uwe Walschus, Anette Kob, Jens-Wolfgang Pissarek, Michael Schlosser

**Affiliations:** ^1^Department of Medical Biochemistry and Molecular Biology, University Medical Center Greifswald, Greifswalder Straße 11c, 17495 Karlsburg, Germany; ^2^Department of Surgery, University Medical Center Greifswald, Ferdinand-Sauerbruch-Straße, 17475 Greifswald, Germany; ^3^MBP Medical Biomaterial Products GmbH, Lederstraße 7, 19306 Neustadt-Glewe, Germany

## Abstract

Two cross-linked acellular porcine dermal collagen matrices (Permacol and NRX) were implanted into rats and the acute and chronic local inflammatory tissue reactions were investigated after 7, 14, 28, and 112 days. Both membranes were stable in vivo for up to 112 days. All investigated immune cell populations (CD68+ macrophages, CD163+ macrophages, T lymphocytes, MHC class II positive cells, mast cells, and NK cells) were present. Their amount decreased significantly over time compared to day 7 after implantation. A change from an acute to a chronic inflammation and an associated shift from proinflammatory M1-like to anti-inflammatory M2-like macrophages were observed. In the early phase there was a significant correlation of T cells to CD68+ (M1-like) macrophages, whereas in the chronic phase T lymphocytes were positively correlated with CD163+ (M2-like) macrophages. The material NRX showed an enhanced inflammatory reaction in comparison to Permacol possibly caused by material characteristics such as a twofold higher thickness of the membrane, roughness, and water absorption capacity. Nevertheless, a more pronounced regenerative process as, for example, indicated by nestin expression demonstrated its possible suitability for applications as wound repair material.

## 1. Introduction

Biological scaffold materials like porcine and bovine collagen are frequently used in reconstructive and regenerative medicine. Collagen, a major component of the extracellular matrix, is known to act as a matrix which mediates the migration and adhesion of cells as well as subsequent vascularization and formation of connective tissue [1]. Due to its potential for tissue regeneration, it is used in different biomedical applications [2]. Nevertheless, besides the desired effect these materials cause a more or less severe local inflammatory reaction after implantation. Among other factors the degree of this reaction depends on the structure and functional characteristics of the implanted material. During the first acute phase of wound repair and inflammation the cellular reaction is characterized by an influx of neutrophils and tissue derived monocytes into the damaged area. While in general the neutrophils stay for only a few days, monocytes transform into macrophages and are the predominant regulatory cell type during the subsequent period of inflammation. Besides removing damaged tissue, cell debris and foreign bodies by phagocytosis they release cytokines and other important growth factors regulating the further reaction.

The acute phase of inflammation persists for several days. If there is foreign material in the wound which cannot be destroyed or phagocytosed a chronic phase of inflammation follows which is characterized by the persistent presence of lymphocytes and macrophages. The macrophages attract fibroblasts building collagen in larger quantities leading to the formation of a fibrous capsule and the occurrence of foreign body granuloma associated with the presence of multinucleated foreign body giant cells generated by macrophage fusion. Macrophages also seem to play a pivotal role in the transition between inflammation and repair [3, 4].

In this regard, the concept of macrophage polarization into distinct subpopulations has gained increasing importance in recent years in the field of biomaterials research [5, 6]. Comparable to proinflammatory T_H_1 and anti-inflammatory T_H_2 phenotypes of T lymphocytes as one central component of the adaptive immune system, macrophages can also be differentiated into proinflammatory M1 type and anti-inflammatory M2 type. The mode of differentiation depends on their interactions with other immune cells via specific surface receptors and their soluble secretory factors like cytokines. In the context of biomaterials, the switch from proinflammatory M1 macrophages, mainly responsible for biodegradation and phagocytosis, to anti-inflammatory M2 macrophages, acting in wound healing and tissue repair, is essential for long-standing success of the implant.

Acellular collagen matrices are used for hernia repair and other medical applications. For instance, the suitability of Permacol (Covidien Deutschland GmbH, Neustadt/Donau, Germany) based on porcine collagen for surgical purposes was verified in different in vitro and in vivo studies, demonstrating stability in vivo and only a mild chronic inflammation [7–11].

The aim of the present study was to compare the local inflammatory reaction after implantation of two different porcine collagen matrices into rats and to differentiate between the acute and the chronic phase of inflammation. For this, simultaneous intramuscular implantation of the two matrices into the neck musculature of rats was chosen as used in previous studies for comparative evaluation of the acute and chronic local inflammatory tissue response for surface-modified implant materials [12–16]. For the present study, the phase until day 7 was designated as the acute phase of inflammation and the later time points as chronic phase (days 28, 56, and 112). Furthermore, the correlation of M1-like and M2-like macrophages with each other as well as their association with T lymphocytes, NK cells, and the regeneration marker nestin was analyzed during these two phases to get a differentiated insight into implant-associated inflammatory processes and to investigate material-dependent differences.

## 2. Material and Methods

### 2.1. Implants

Two acellular porcine dermis collagen matrices, Permacol (Covidien Deutschland GmbH, Neustadt/Donau, Germany) and NRX (Non-Resorbable Xenoderm; MBP, Medical Biomaterial Products GmbH, Neustadt-Glewe, Germany), were used for the in vivo study. While both of these materials were produced from porcine dermis cross-linked by noncalcifying hexamethylene diisocyanate using proprietary processes of the respective manufacturer, there are also differences regarding their preparation. Most importantly, Permacol was purified with organic solvents and dried by acetone rinsing. In contrast, no organic solvents were used for purification of NRX, and dehydration was done by freeze drying. Before implantation dry and wet weight of 5 × 5 mm pieces of both membranes was determined and 5 *μ*m slices were cut with a Cryomicrotome 2800 Frigocut N (Reichert-Jung, Nussloch, Germany), mounted on slides and stained with Haematoxylin/Eosin and Gomori's trichrome. Using digital images of the stained material (objective lens magnification 20×), thickness of the membranes and percentage of fibres of the whole membrane area were determined by light microscopy (Olympus CX41 with digital camera DP20, Olympus, Hamburg, Germany) using the image analysis software ImageJ v1.44 (US National Institutes of Health, Bethesda, MD, USA).

### 2.2. In Vivo Study

Following anaesthesia by intraperitoneal application of a mixture of Rompun (Bayer, Leverkusen, Germany) and Ketamin (Sanofi-Ceva, Düsseldorf, Germany), pieces (5 × 5 mm) of both materials were simultaneously implanted into small tissue pockets carefully cut with a scalpel into the spinotrapezius muscle of 24 male Lewis rats (weight: 360 ± 10 g, age: 100 days; Charles River Laboratories, Sulzfeld, Germany) with a distance between the implants of about 3 cm. The implants were fixed within the respective tissue pockets using non-resorbable Prolene 6-0 suture (Ethicon, Norderstedt, Germany). All animals recovered completely from the implantation within short time and no animal demonstrated any signs of sickness or other problems caused by the surgical procedure. Animals were kept under conventional conditions, fed ad libitum with a standard diet, and had free access to water. Six randomly selected rats were euthanized 7, 28, 56, and 112 days after implantation. The implants with the surrounding tissue were surgically removed, shock frozen in liquid nitrogen, and stored at −70°C until histological investigation.

All animal experiments were conducted in full accordance with the animal protection law of the Federal Republic of Germany in its new version of 1987, with the principles of care for animals in laboratories (drawn up by the National Society for Medical Research) and with the Guidelines for Keeping and Using Laboratory Animals (NIH Publication number 80-23, revised in 1985).

### 2.3. Histology and Immunohistology

Cryosections of 5 *μ*m thickness were prepared from the implants with the surrounding tissue with a Cryomicrotome 2800 Frigocut N (Reichert-Jung, Nussloch, Germany), air dried, and fixed for 10 min in ice-cold acetone. Sections were stained with Haematoxylin and Eosin (HE), Gomori's trichrome, and Toluidine blue according to standard protocols. Immunohistochemical staining of CD68+ (M1-like) macrophages (monoclonal mouse-anti-rat-CD68 antibody ED1), CD163+ (M2-like) macrophages (monoclonal mouse-anti-rat-CD163 antibody ED2), T lymphocytes (monoclonal mouse-anti-rat-TCR*α*/*β* antibody R73), MHC class II positive cells (monoclonal mouse-anti-rat-RT1B antibody OX6)—all antibodies obtained from ABD Serotec (Bio-Rad AbDSerotec GmbH, Puchheim, Germany)—activated NK cells (monoclonal mouse antibody ANK61, Santa Cruz Biotechnology, Inc., Heidelberg, Germany), and the marker nestin for neogenesis of cells (monoclonal mouse-anti-mouse/rat nestin antibody Rat-401, eBioscience Inc., Frankfurt, Germany) was performed according to the respective manufacturer's protocols. The APAAP method with the polyclonal rabbit anti-mouse-immunoglobulin (Z259, Dako DenmarkA/S, Glostrup, Denmark), the mouse monoclonal antibody alkaline-phosphatase-anti-alkaline phosphatase (APAAP, clone AP1B9, Sigma-Aldrich Chemie Gmbh, Munich, Germany), and New fuchsine was used to detect bound antibodies [17, 18]. After short staining of the nuclei with Haematoxylin/Eosin the sections were covered and dried.

### 2.4. Morphometric Evaluation

Digital images of the stained tissue with the implant were captured using a light microscope CX41 (Olympus, Hamburg, Germany) together with a digital colour camera DP20 (1600 × 1200 Pixel, Olympus, Hamburg, Germany) at an objective lens magnification of 10× and evaluated with the image analysis software ImageJ v1.44 (US National Institutes of Health, Bethesda, MD, USA). Thickness of the membranes and the layer containing immune cells surrounding the implant (“reaction layer”) was measured and given as *μ*m. For estimation of the stained area for the different antibodies 5–10 pictures of the implant surrounding tissue were taken and the stained area was measured as percentage of the whole tissue area. The number of foreign body giant cells of five microscopic visual fields (area about 5 mm^2^) in the surrounding of the implant was counted using HE-stained slides and calculated as cells/mm^2^.

### 2.5. Statistical Analysis

Data are either given as median and interquartile range or, where applicable, as mean and standard deviation. For comparison of samples, the Mann-Whitney test was used for non-paired data sets, including comparison between different experimental days, and the Wilcoxon signed rank test was used for paired data sets, including comparison between materials on the same experimental day. Among the examined days, the acute inflammatory phase was considered to last until day 7 while the later time points (days 28, 56, and 112) constituted the chronic phase. Furthermore, Pearson's correlation analysis was used to analyze associations between the different immune cell populations investigated in the acute and chronic phase of inflammation.

## 3. Results

### 3.1. Membrane Characteristics before Implantation

**Figure 1:**
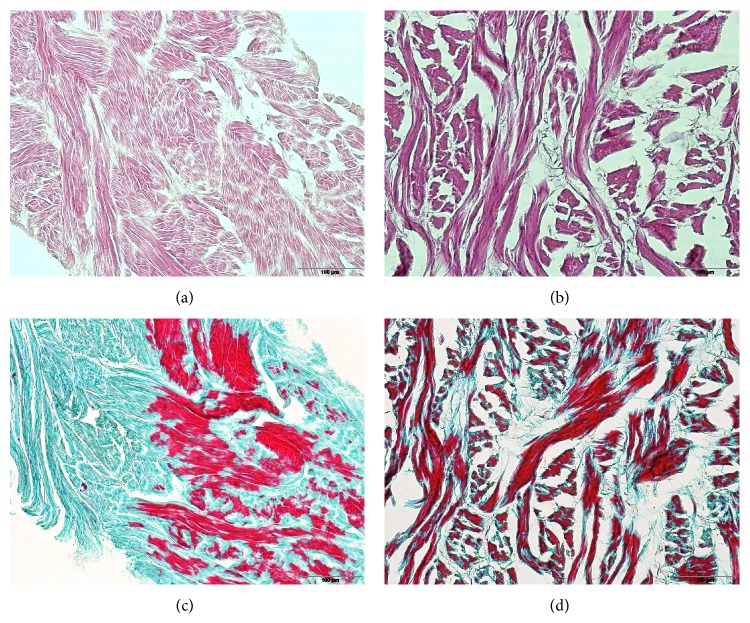
Haematoxylin/Eosin ((a) and (b)) and Gomori's trichrome ((c) and (d)) staining of Permacol ((a) and (c)) and NRX ((b) and (d)) membranes before implantation (objective lens magnification 20×). Porcine collagen fibres are stained green ((c) and (d)).

In Haematoxylin/Eosin and Gomori's trichrome stained slices of both membranes before implantation, the fibres and the green stained connective tissue within the membranes were clearly visible. The structure of NRX was not as dense as that of Permacol and the content of connective tissue appeared to be less (Figure 1). This was confirmed by the morphometric estimation of fibre density of both membranes (Table 1). Additionally, the NRX membrane was characterized by a two-fold higher thickness and water adsorption capacity compared to Permacol (Table 1).

### 3.2. Histological Evaluation of Membranes following Implantation

The histological investigation of the implant site showed that all implants were stable up to 112 days in vivo. The significant difference in membrane thickness of Permacol and NRX which was observed before implantation (Table 1) persisted over the whole period of investigation. Nevertheless, the thickness of membranes was significantly reduced by about 20% after 112 days in vivo compared to the time before implantation (Permacol: 333.4 ± 5.2 *μ*m versus 263.9 ± 21.5 *μ*m, *P* < 0.01, and NRX: 631.2 ± 19.5 *μ*m versus 525.8 ± 37.5 *μ*m, *P* < 0.0001).

**Figure 2:**
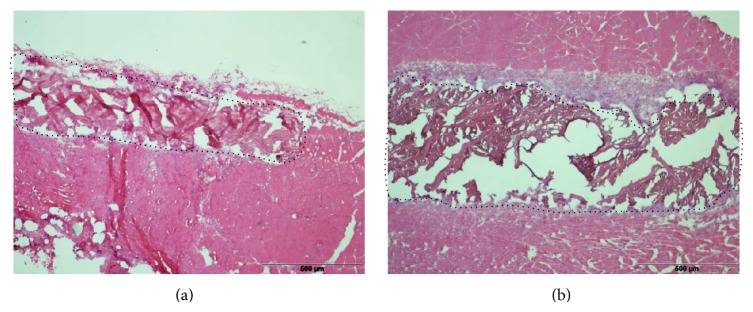
Haematoxylin/Eosin-stained sections of Permacol (a) and NRX (b) implant 112 days after implantation; dotted line shows the periphery of the implant (objective lens magnification 4×).

The membranes were encapsulated by a continuously growing layer of fibrotic tissue and in the direct surrounding of the implants a zone with inflammatory cells was detectable (Figure 2).

**Figure 3:**
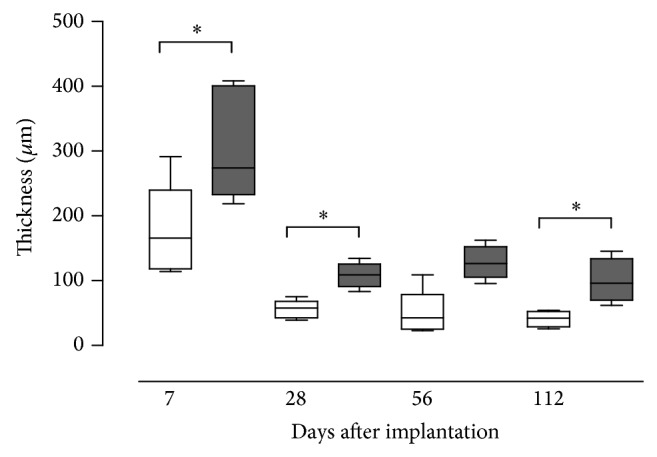
Thickness of the “reaction layer” after implantation of Permacol (white boxes) and NRX (grey boxes). Boxes represent median and interquartile range, whiskers minimum and maximum values. ^*^ *P* < 0.05 Wilcoxon signed rank test.

A sustained inflammatory reaction was observed at the implant site for both materials at any investigated time point. In the early phase after implantation the surrounding of the implants appeared oedematous and contained many inflammatory cells. In the later phase it became more structured and fibrotic. The thickness of this “reaction layer” surrounding the implant decreased with time for both materials but was significantly higher for NRX implants compared to Permacol implants at days 7, 28, and 112 (*P* < 0.05, Figure 3).

*Foreign Body Giant Cells*. The number of foreign body giant cells was significantly higher in NRX implants than in Permacol implants on days 28 and 56, whereas at days 7 and 112 after implantation no differences were observed (Table 2). For Permacol implants the number of giant cells decreased significantly (*P* < 0.05) at days 56 and 112 compared to day 7; for NRX implants there were significantly (*P* < 0.05) lower numbers of foreign body giant cells at day 112 compared to day 28.

### 3.3. Morphometric Immunohistochemical Evaluation

**Figure 4:**
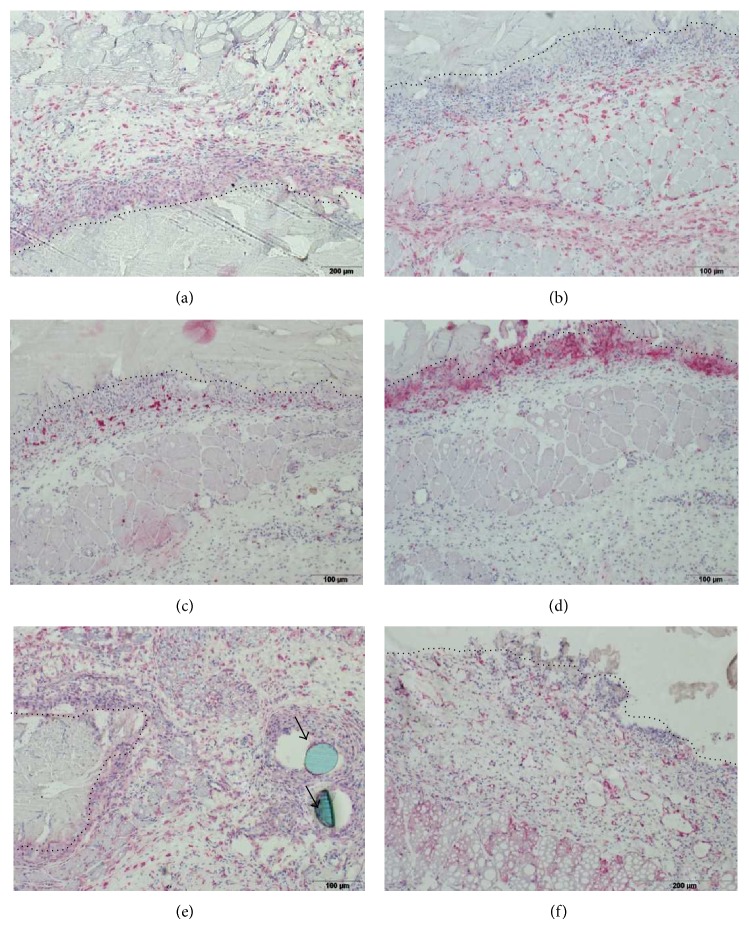
Exemplary demonstration of immunohistochemically stained cells surrounding Permacol 7 days after implantation: (a) CD68+ (M1-like) macrophages; (b) CD163+ (M2-like) macrophages; (c) T lymphocytes; (d) NK cells; (e) MHC class II positive cells; (f) nestin positive cells. Dotted line indicates border between implant and surrounding tissue, arrows (e) show nonabsorbable surgical suture. The objective lens magnification was 10× for all images.

All types of investigated immune cells were detectable; exemplarily shown are immunohistochemical stained sections of peri-implant tissue after implantation of Permacol at day 7 (Figure 4).

**Figure 5:**
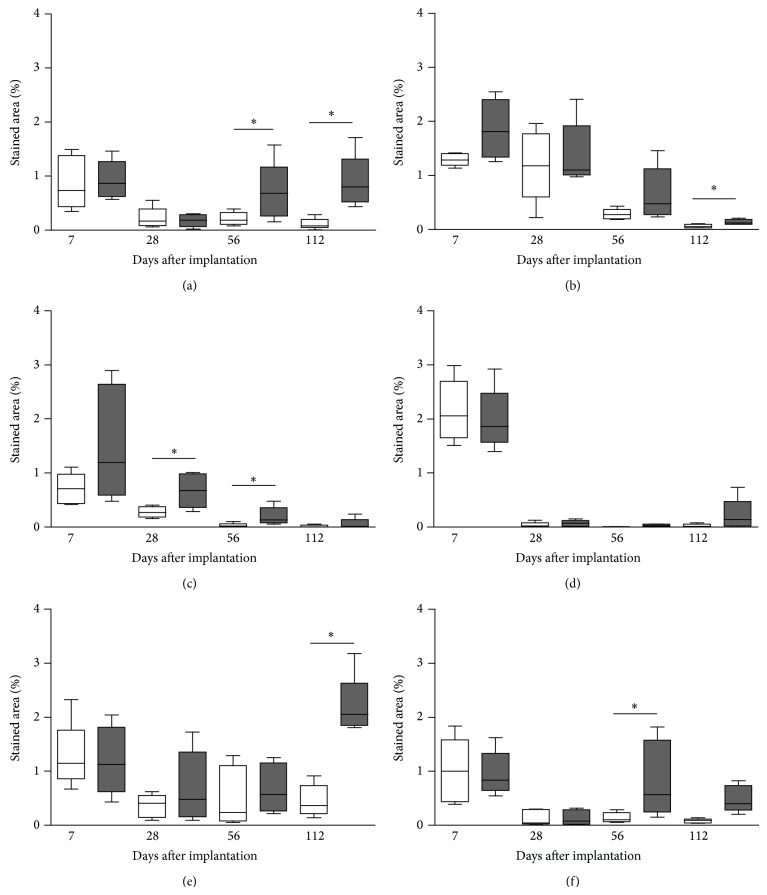
Percentage of stained area for different immune cells in the peri-implant tissue after implantation of Permacol (white boxes) and NRX (grey boxes). Boxes represent median and interquartile range, whiskers minimum and maximum values. ^*^ *P* < 0.05, nonparametric Wilcoxon signed rank test. (a) CD68+ (M1-like) macrophages; (b) CD163+ (M2-like) macrophages; (c) T lymphocytes; (d) NK cells; (e) MHC class II positive cells; (f) nestin positive cells.

*CD68+ (M1-Like) Macrophages*. The positively stained area for CD68+ (M1-like) macrophages for both materials was about 1% (median) at day 7 after implantation and decreased to about 0.2% at day 28 (Figure 5(a)). At days 56 and 112 after implantation the amount of cells significantly increased for the NRX samples in comparison to Permacol to a median of 0.68% (interquartile range IQR: 0.26–1.17) versus 0.19% (0.11–0.33) and 0.80% (0.52–1.31) versus 0.08% (0.04–0.20), respectively (*P* < 0.05).

*CD163+ (M2-Like) Macrophages*. The amount of CD163+ (M2-like) macrophages did not differ between the samples up to day 56 after implantation (Figure 5(b)). Only on day 112, a significant (*P* < 0.05) increased positively stained area in NRX samples with a median of 0.12% (IQR: 0.09–0.19) versus 0.05% (0.03–0.09) for Permacol was observed.

*T Lymphocytes*. At day 7 after implantation the amount of T lymphocytes was about 1% for both materials (Figure 5(c)). At day 28 and 56 a significant (*P* < 0.05) increase in the cell amount for NRX implants in comparison to Permacol was found amounting to a median of 0.68% (IQR: 0.36–0.99) versus 0.27% (0.18–0.38) and 0.14% (0.08–0.36) versus 0.02% (0.005–0.06), respectively.

*Natural Killer Cells*. For the NK cells, a maximum at day 7 and a pronounced decline afterwards was observed for both materials (Figure 5(d)). There was no significant difference between Permacol and NRX on any experimental day. On day 112, the NRX samples exhibited a larger variation due to an increased cell number for some individual animals.

*MHC Class II Positive Cells*. The amount of MHC class II positive antigen-presenting cells demonstrated a slight but nonsignificant decreasing trend over time for both materials (Figure 5(e)). In comparison between NRX and Permacol, both materials exhibited a similar amount with the exception of day 112 after implantation with a significantly enhanced (*P* < 0.05) stained area for NRX with a median of 2.06% (IQR: 1.85–2.63) compared to Permacol 0.37% (0.22–0.73).

*Nestin Expression*. The nestin positively stained area was about 1% on day 7 for both materials but decreased significantly over time (Figure 5(f)). The NRX samples had a significant higher level of nestin expression on day 56, with a median of 0.57% (IQR: 0.24–1.58) for NRX versus 0.11% (0.07–0.24) for Permacol (*P* < 0.05). On day 112, a similar trend was observed for both materials.

*Mast Cells*. The number of mast cells stained with Toluidine blue was low throughout the study course and not significantly different between the tested materials over the whole observation period (data not shown).

### 3.4. Correlation Analysis

**Figure 6:**
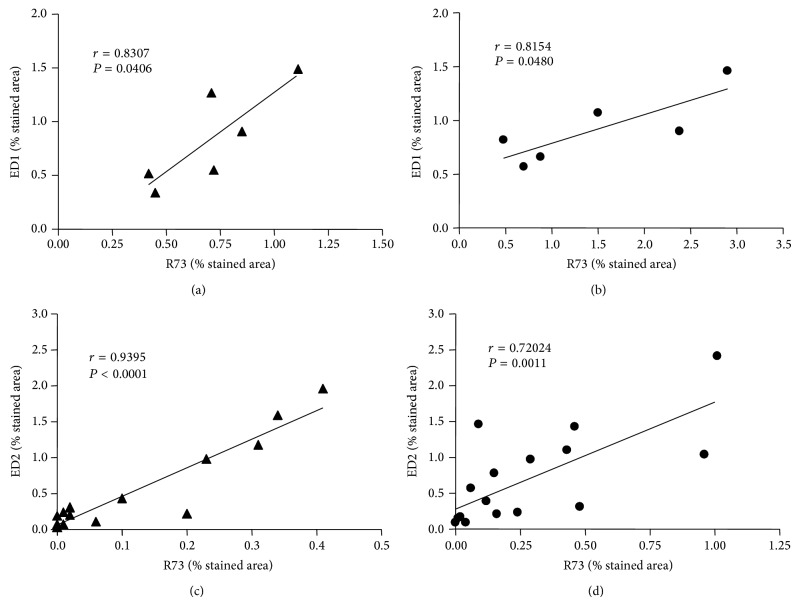
Correlation analysis between stained area of T cells (R73) and CD68+ (M1-like) macrophages (ED1) as well as CD163+ (M2-like) macrophages (ED2) for Permacol (a, c) and NRX (b, d) in the acute (experimental day 7; (a), (b)) and chronic phase (experimental days 28, 56, and 112; (c), (d)) of inflammation. Data are given as Pearson's *r* and *P* values.

To analyze interactions between the response of the different cell types examined during different phases of inflammation, a correlation analysis over the results of all animals on the respective experimental days was performed (Table 3). In the acute phase of inflammation (day 7 after implantation), a significant positive correlation between the T lymphocytes and the proinflammatory CD68+ (M1-like) macrophages was found for both materials (Figures 6(a) and 6(b)), whereas this was not found in the chronic phase of inflammation on experimental days 28, 56, and 112. In contrast, the T lymphocytes demonstrated a significant positive correlation (*P* < 0.0001 for Permacol and *P* = 0.0011 for NRX) with the anti-inflammatory CD163+ (M2-like) macrophages during the chronic phase of inflammation (Figures 6(c) and 6(d)), whereas this was not seen in the acute phase of inflammation. Furthermore, a significant positive correlation of CD68+ (M1-like) macrophages with CD163+ (M2-like) macrophages (*P* = 0.0197) as well as CD163+ (M2-like) macrophages and the level of nestin expression (*P* = 0.0434) was observed for Permacol implants in the chronic phase of inflammation. Additionally, the cellular response for these implants was characterized by a significant positive correlation between the IL2R-positive cells and the nestin expression in the chronic phase (*P* = 0.0071). In comparison to that, for the NRX implants a negative correlation of CD68+ (M1-like) macrophages with CD163+ (M2-like) macrophages was found in the chronic phase of inflammation (*P* = 0.0377). Also, no correlation was observed for these implants between either the CD68+ (M1-like) macrophages or the IL2R-positive cells and the nestin expression.

## 4. Discussion

Biological scaffold materials derived from extracellular matrix (ECM) of mammals, extensively studied regarding their structural properties, mechanical behaviour, and degradation characteristics in vitro and in vivo, have been successfully used in several medical applications [19]. On behalf of their use for medical purposes these biomaterials should cause only low inflammatory responses and have no or negligible antigenicity and a good stability in vivo. Furthermore, the quality and stability of surgical implants used as a scaffold or for wound repair depends on the balance between implant degradation and the growth of native tissue.

In the present study it was demonstrated that Permacol as well as NRX membranes, both based on porcine collagen, were stable up to 112 days in vivo after i.m. implantation in Lewis rats with a decrease of about 20% of their original thickness over this time. Possible explanations for this might be biodegradation and/or shrinking of the material. Recently, it has been shown that cross-linked Permacol was more stable in vivo than non-cross-linked material such as Strattice (LifeCell Corporation) [20]. Thus the biological stability of the materials investigated in the present study can also be explained by their partial cross-linking.

While an inflammatory reaction as well as an encapsulation was observed at the implant site for both materials, the encapsulation layer was thinner for Permacol compared to NRX. This might be either due to the surface characteristics of the different materials or the about twofold higher initial thickness and ratio of wet/dry weight of NRX in comparison to Permacol. It is therefore conceivable that the less compact structure of NRX offers more contact points for immune cells. This is in accordance with the observed higher macrophage response in the surrounding tissue of NRX implants in the later chronic phase after implantation (days 56 and 112). Also, the higher amount of fibrous tissue found around the NRX implant is in line with these observations since macrophages secrete fibronectin as a chemotactic factor attracting fibroblasts and other growth factors for ECM production.

Based on their specific function macrophages can be subdivided similar to T lymphocytes into a “classically activated” proinflammatory (M1) and an “alternatively activated” anti-inflammatory (M2) phenotype [21–23]. M1 macrophages act as effector cells in T_H_1 cellular immune response, whereas M2 macrophages are involved in wound healing, tissue repair, and immunosuppression [5, 6, 24, 25]. It is known that cytokines of T_H_1 lymphocytes like interferon *γ* (IFN*γ*) and interleukin-1*β* (IL-1*β*) induce a “classical” activation profile (M1) of macrophages. The initial phase of inflammation is characterized by a typical T_H_1 response [26, 27]. T lymphocytes also play a regulatory role in inflammation and wound healing. Cytokines and chemokines secreted by T_H_2 cells such as IL-4, IL-10, and IL-13 affect polarization of macrophages, inducing an “alternative” activation program which results in M2-type macrophages involved in wound healing and activation of B-cells followed by a humoral immune reaction [25, 28–30].

It should be noted that any histological approach for differentiation of M1 and M2 macrophages is in principle restricted by the fact that there are no known markers with exclusive expression on either one of these macrophage subtypes. However, the antibody ED1 which was used in the present study recognizes the antigen CD68 which is expressed by monocytes and inflammatory macrophages [31, 32]. Thus, while CD68 is not an M1 marker in a strict sense and might, for example, also be expressed on nondifferentiated macrophages, its expression can be reasonably assumed to reflect the level of M1 macrophage activity, at least for the purpose of comparing different materials under otherwise similar conditions. The antibody ED2 which was used in this study recognizes the antigen CD163. This marker has been shown by other authors to be highly indicative of polarization of macrophages to the anti-inflammatory M2 type [5]. Therefore, in the present study cells stained positive with the antibody ED1 were designated as proinflammatory CD68+ (M1-like) macrophages and cells stained positive with the antibody ED2 as anti-inflammatory CD163+ (M2-like) macrophages.

The results presented here indicate that both types of macrophages were involved in the inflammatory response after implantation of porcine collagen matrices. The number of proinflammatory CD68+ (M1-like) macrophages declined with time after implantation, but for the NRX implants in the late phase of inflammation their number increased again, caused by different material properties. The number of foreign body giant cells (FBGC) which are formed by fusion of macrophages decreased from day 7 to day 112 for Permacol while a temporary increase with its maximum on day 28 followed by a delayed decline was observed for NRX. Except for day 7, the number of FBGC was significantly higher for NRX than for Permacol. Regarding cellular interactions, a significant positive correlation between CD68+ (M1-like) macrophages and T lymphocytes was observed in the acute phase of inflammation for both materials which was not seen in the chronic phase. It can be assumed, along with published data [26, 27], that the positive correlation between T cells and CD68+ (M1-like) macrophages observed in the present study was predominantly caused by T_H_1 cells.

At day 28 after implantation the positively stained area of CD163+ (M2-like) macrophages was higher than that of CD68+ (M1-like) macrophages. This shows a switch of CD68+ (M1-like) macrophages dominating in the early phase after muscle injury (implantation) to CD163+ (M2-like) macrophages in the chronic phase and is in accordance with recent findings [27]. Also, the amount of CD163+ (M2-like) macrophages was significantly higher for NRX compared to Permacol. Barth et al. [33] described that a rough surface of a material promoted polarization to the M2 phenotype involved in wound repair and is in accordance with differences in material properties.

In the late phase after implantation a striking positive correlation between T lymphocytes and CD163+ (M2-like) macrophages was found for both materials, whereas this was not the case in the acute phase of inflammation. It can be assumed that these T cells were of T_H_2 phenotype and involved in humoral immunity and coordination of immune response to the implanted material. Examining the antigen-presenting MHC class II antigen positive cells we found similar results for both materials; only at day 112 their amount was significantly higher for NRX implants. Additionally, the proinflammatory IL-2 plays a critical role in differentiation of T cells [34]. Concerning the increasing nestin positive area for NRX and its correlation to the IL-2 receptor expression in the late phase of inflammation it could be speculated that this might cause a bigger stimulus for the body and could result in a more pronounced inflammatory reaction. This might be on the one hand a more intensive immune reaction, possibly combined with the generation of specific antibodies against the porcine collagen matrix; on the other hand it could reflect a higher regeneration rate of the implant surrounding tissue, which might be desired and especially useful for instance when the material is used for wound repair. Carlson et al. [35] found that due to the cross-linked structure Permacol showed a delayed remodelling capacity compared to a non-cross-linked material. A limited fibrovascular ingrowth into Permacol was also described by Hammond et al. [7].

In summary, after implantation of Permacol and NRX membranes into rats an inflammatory reaction with different, material-dependent characteristics was observed which changed from an acute to a chronic phase. Therefore, the cross-linked NRX, despite a moderately stronger inflammatory response, seems to be suitable for wound repair since an increased regeneration was observed in the chronic phase of inflammation. Nevertheless, there are also indications for humoral immune reactions such as generation of specific antibodies against porcine collagen as macrophages, antigen presenting cells, and T lymphocytes were found to be involved in the acute and chronic inflammatory phase after implantation. However, this question remains open and has to be answered in further investigations.

## Figures and Tables

**Table 1:** Membrane characteristics before implantation (data are mean of 20 different measurements ± standard deviation).

	Permacol	NRX
Thickness	333 ± 5 *µ*m	631 ± 19 *µ*m
Fibre density	66.5 ± 1.7%	52.2 ± 1.4%
Ratio wet/dry weight	1.9	3.7

**Table 2:** Number of foreign body giant cells after implantation of Permacol and NRX membranes. Wilcoxon signed rank test.

Material	Foreign body giant cells (cells/mm^2^)			
	Day 7	Day 28	Day 56	Day 112
Permacol	0.60 ± 0.26 (6)	0.23 ± 0.12 (6)	0.03 ± 0.03 (6)	0 (5)
NRX	1.05 ± 0.36 (6)	1.90 ± 0.37 (6)	1.65 ± 0.57 (6)	0.42 ± 0.25 (5)
Wilcoxon signed rank test	*P* = 0.4375	*P* = 0.0313	*P* = 0.0313	*P* = 0.5000

**Table 3:** Correlation analysis between stained area of CD68+ (M1-like) macrophages (ED1) and CD163+ (M2-like) macrophages (ED2), T lymphocytes (R73), regenerative cells (Rat-401), and activated Il-2R-positive cells (OX39) for Permacol and NRX differentiated into the acute (experimental day 7) and chronic (experimental days 28, 56, and 112) phase of inflammation. Data are given as Pearson's *r* and *P* values.

Antibodies	Permacol				NRX			
	Acute phase		Chronic phase		Acute phase		Chronic phase	
	*r*	*P*	*r*	*P*	*r*	*P*	*r*	*P*
ED1 versus ED2	0.2691	0.6060	**0.5590**	**0.0197**	−0.4315	0.3930	**−0.5073**	**0.0377**
R73 versus ED1	**0.8307**	**0.0406**	0.4629	0.0531	**0.8154**	**0.0480**	−0.3574	0.1454
R73 versus ED2	0.1209	0.8195	**0.9395**	**<0.0001**	−0.3845	0.4516	**0.7202**	**0.0011**
Rat-401 versus ED1	0.0270	0.9595	**0.4809**	**0.0434**	−0.1489	0.8111	0.1708	0.5812
Rat-401 versus OX39	0.5760	0.2315	**0.6111**	**0.0071**	0.0645	0.9179	0.2922	0.2551
